# Supplementary material for: Collective directional migration drives the formation of heteroclonal cancer cell clusters
Source: Mol Oncol. 2023 Jan 28;17(9):1699–725. doi: 10.1002/1878-0261.13369 (PMC10483614; doi:10.1002/1878-0261.13369)
Supplement: Supplementary file 1 — Fig. S1. Nonaggregating cell lines. Fig. S2. CDM in different matrices. Fig. S3. Proliferative dynamics. Fig. S4. Toxicity of mitomycin treatments. Fig. S5. Cytoskeletal perturbation of CDM. Fig. S6. Toxicity of downstream inhibitors. Fig. S7. Effect of conditioned media on migration. Fig. S8. Cross‐reactivity of conditioned media. Fig. S9. Heteroclonal aggregation of breast cancer lines. Table S1. List of cell lines used in this paper. Table S2. List of ligand–receptor couples with references. [file MOL2-17-1699-s002.pdf]

Supplementary Material for:
Collective directional migration drives the formation of heteroclonal cancer cell clusters

Miriam Palmiero[1,2], Isabel Cantarosso[1,2], Laura di Blasio[1,2], Valentina Monica[1,2], Barbara Peracino[3], Luca Primo[1,2,*] and Alberto Puliafito[1,2,*]

[1] Candiolo Cancer Institute, FPO - IRCCS, Str. Prov. 142, km 3.95, 10060 Candiolo, Italy.
[2] Department of Oncology, University of Turin, 10060 Candiolo, Italy
[3] Department of Clinical and Biological Sciences, University of Turin, San Luigi Hospital, 10043 Orbassano, Italy
[*] these authors contributed equally
Corresponding author: alberto.puliafito@ircc.it, Phone number: +39 011 993 3505;

Supplementary movies and full size figures can be found at the following link:

https://osf.io/meygs/

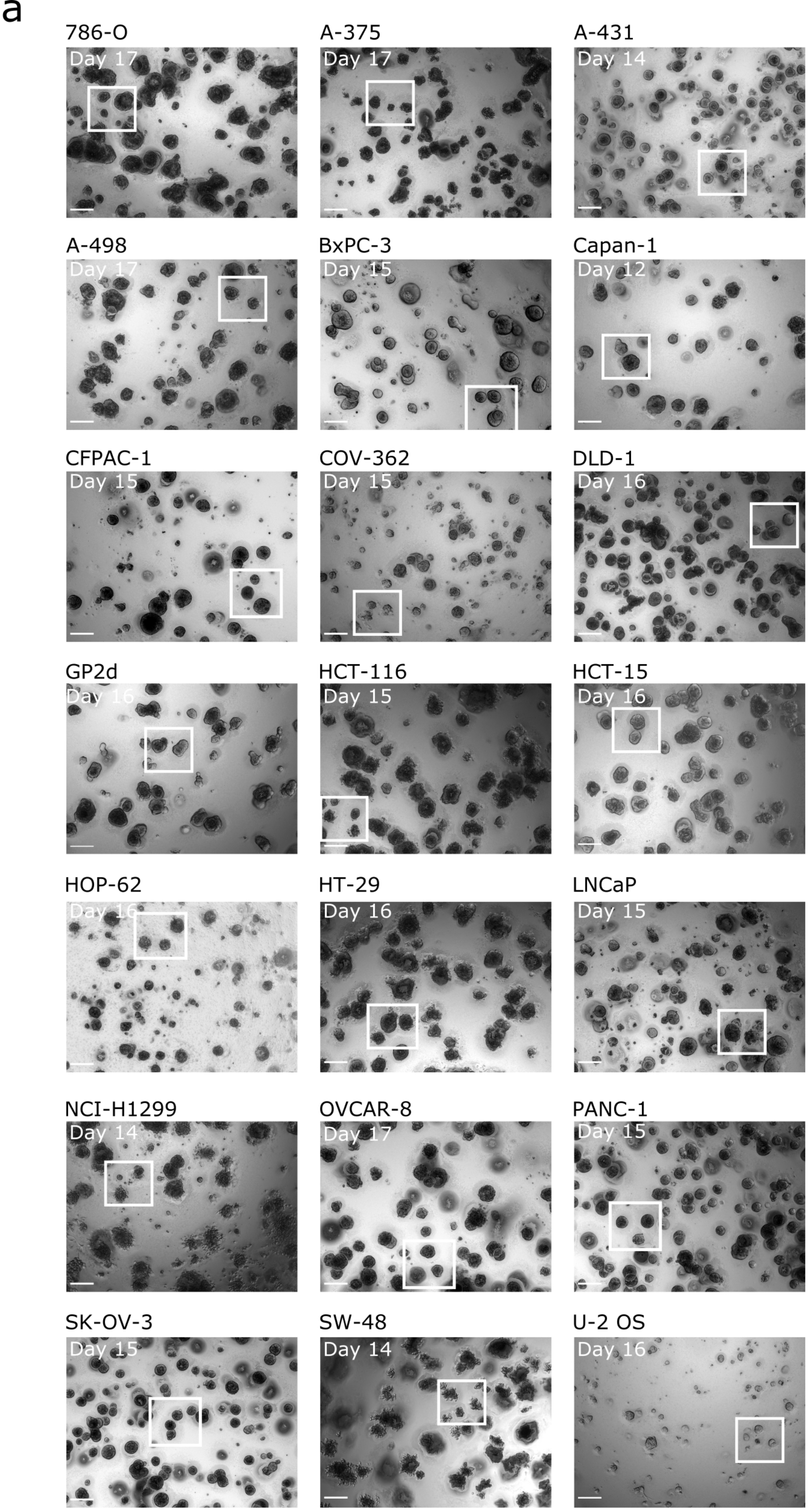

Supplementary figure 1: Non aggregating cell lines (a) Representative CC lines, derived from different tissues of origin, were used in our aggregation assay showing a non-aggregating phenotype. Cells were seeded as single-cell suspension in Matrigel and imaged by means of time-lapse bright-field microscopy for several weeks. One representative snapshot for each cell line at the end of the aggregation assays (time-points are indicated on the top-left corner of the pictures) is shown. White squares point out clusters that just grow without touching each other. Scale bar: 200 μm.

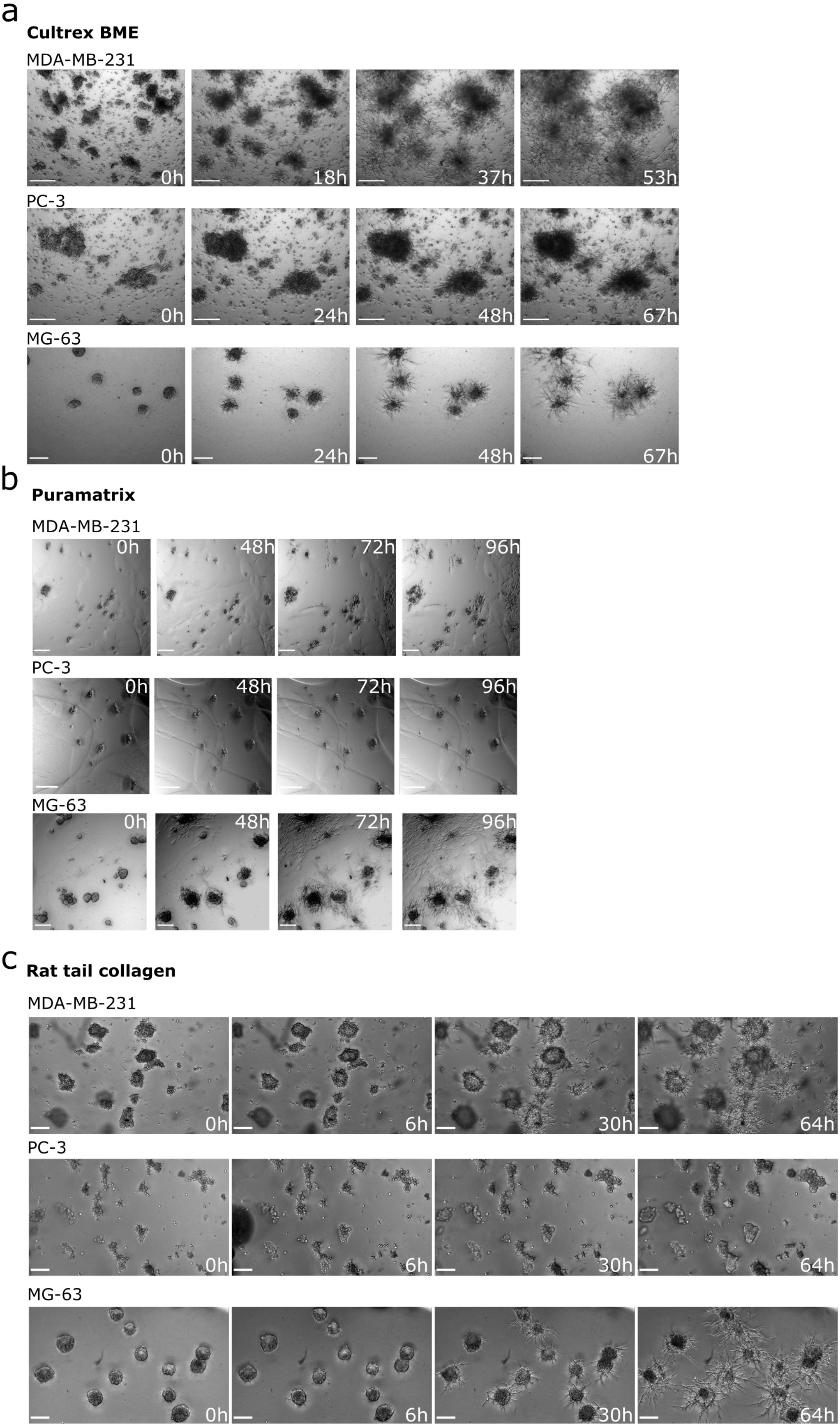

Supplementary figure 2: CDM in different matrices Representative snapshots of preformed spheroids embedded in different hydrogels. **(a)** Cultrex BME. In this hydrogel pre-formed spheroids behave analogously to what observed in Matrigel. **(b)** Corning Puramatrix. Here we found spheroids to be much less protrusive and non-migratory. **(c)** Roche Type I Rat-tail Collagen. Spheroids grown in collagen are generally more protrusive, displaying multicellular outgrowths collectively invading the surrounding matrix. No bulk spheroid movement is observed in this case. Scale bar: 200 μm.

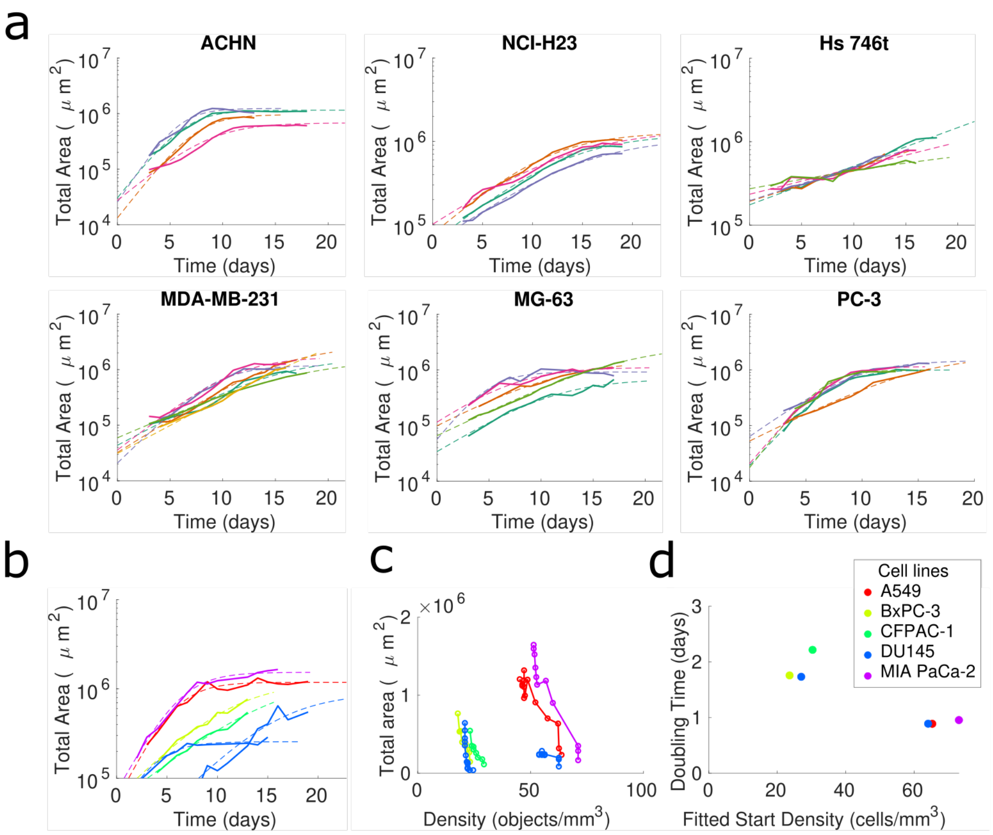

Supplementary figure 3: Proliferative dynamics (a,b) Timeseries of the total area for each density of the indicated aggregating cell lines (a) and non-aggregating cell lines (b), indicated in the title of the plot or in the legend in panel (d). Dashed lines represent the fit with a saturating exponential. **(c)** Scatter-plot of the total area vs density for non-aggregating cell lines. **(d)** Doubling times for non-aggregating cell lines plotted against the starting density.

a

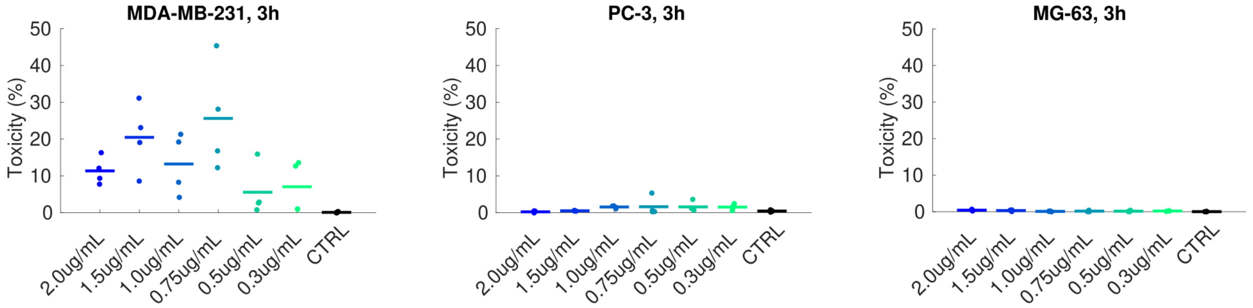

Supplementary figure 4: Toxicity of mitomycin treatments (a) The level of toxicity for each concentration of mitomycin is evaluated by assessing the cumulative number of apoptotic events normalized by the number of nuclei at the beginning of the experiment, marked by a fluorescent signal triggered by Cell Event Casp3/7 over the course of the timelapse (19 hours).

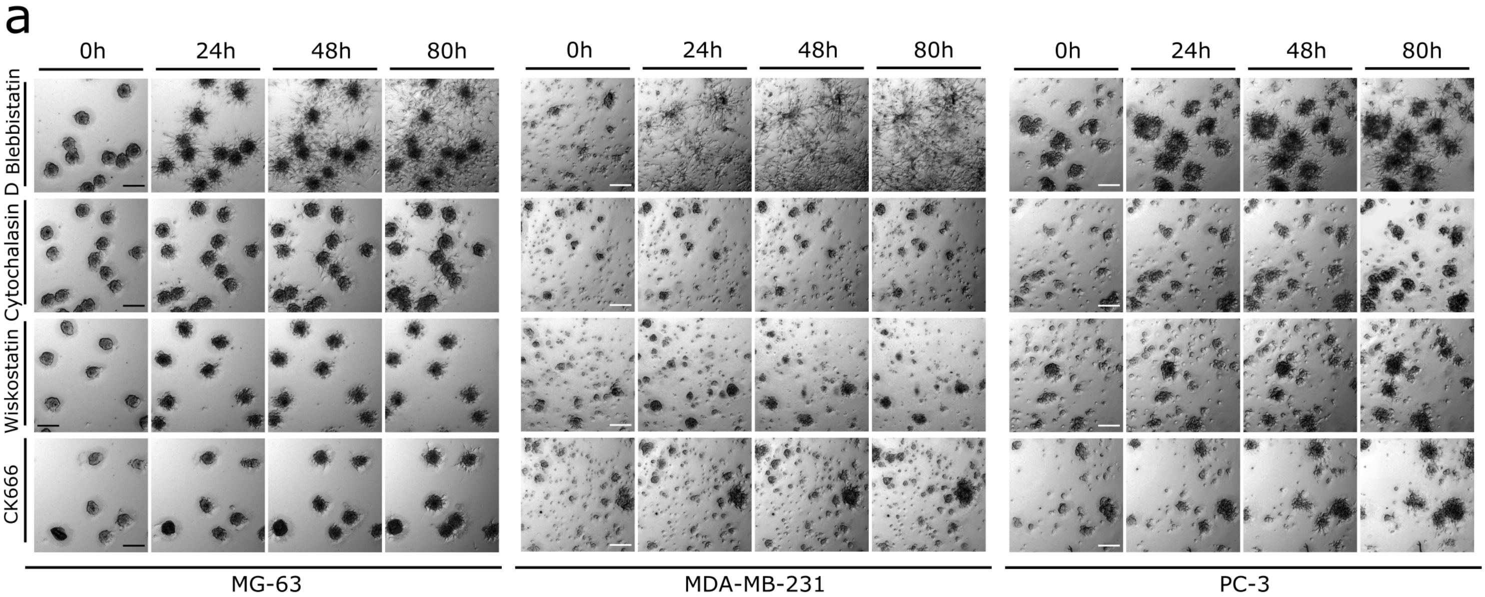

Supplementary figure 5: Cytoskeletal perturbation of CDM (a) Pre-formed spheroids of three representative aggregating cell lines were embedded in Matrigel and observed by means of bright-field microscopy for several days (From left to right: MG-63; MDA-MB-231 and PC-3). Pictures taken at 0, 24, 48, and 80 hours after seeding are shown. The first row of each set of images shows spheroids treated with 100 µM Blebbistatin, the second-row spheroids treated with 1 µM Cytochalasin D, the thirdrow spheroids treated with 10 µM Wiskostatin and the last row spheroids treated with 100 µM CK666. Seeding density: 2,5 spheroids/mm3. Scale bar: 200 µm.

a

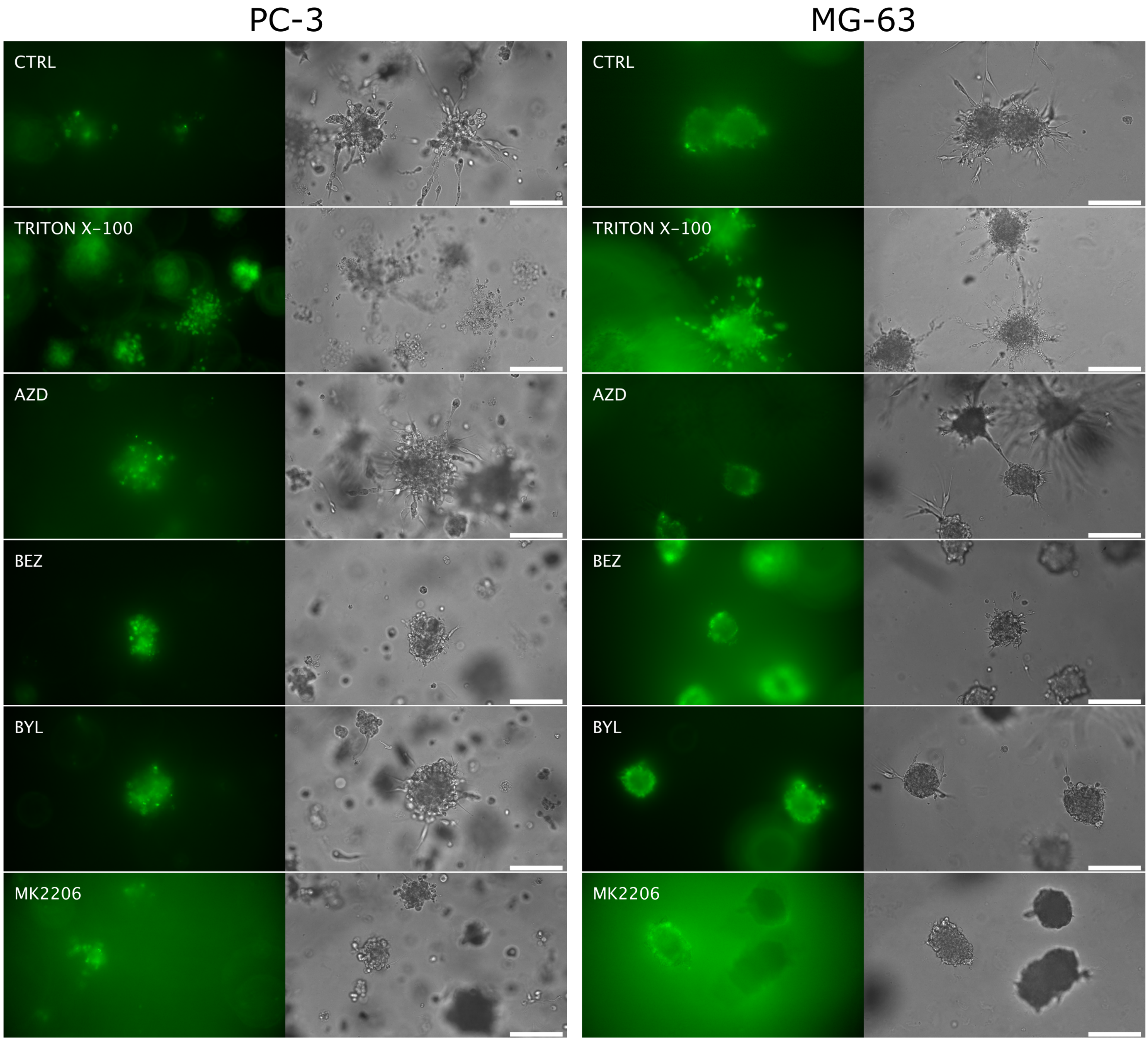

b

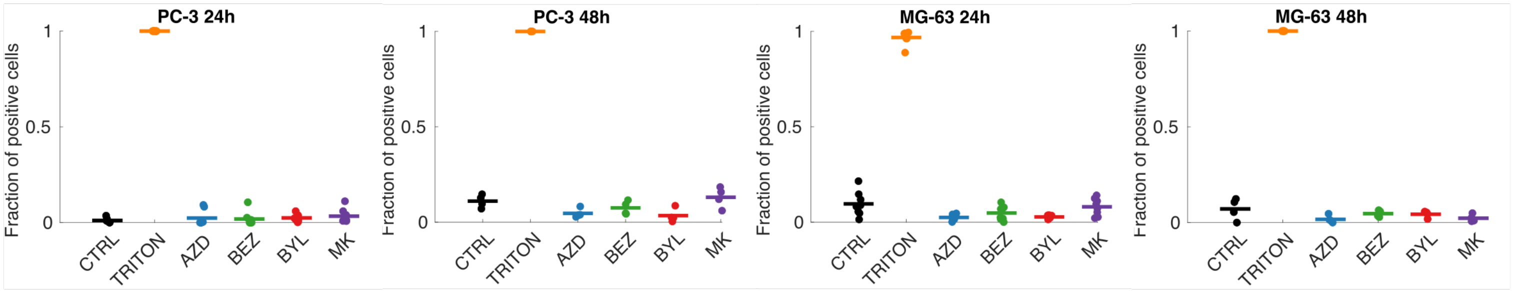

Supplementary Figure 6: Toxicity of downstream inhibitors (a) Toxicity effects of downstream effector inhibition on PC-3 and MG-63 cell lines. Green indicates the signal coming from CellToxGreen, and grayscale images are brightfield. The concentrations used for the inhibitors were: MK2206 3mM; BYL719 3mM; BEZ235 300nM; AZD644 0.5mM. Scale bar: 200μm. **(b)** Quantification of the impact of treatment on cells cultured in 2D expressed as fraction of CellToxGreen positive cells over the total at 24 and 48 hours.

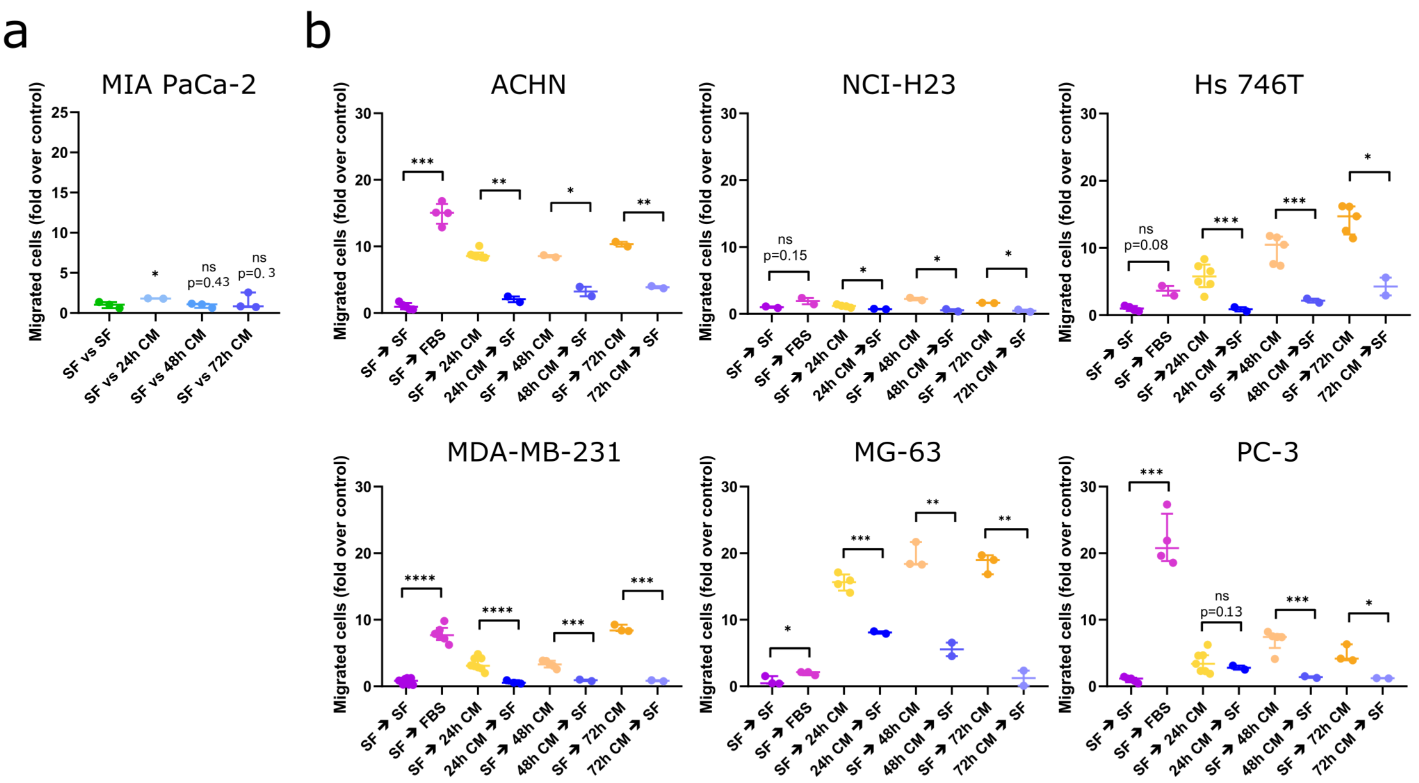

Supplementary figure 7: Effect of conditioned media on migration (a) Transwell assay performed with MIA PaCa-2 cells. As in Fig. 7, the plots report the migrated cells (fold over control) for conditioned media collected at different times. The data indicate that MIA PaCa-2 are not able to migrate toward their own conditioned media. Each point on the plot represents data coming from a whole membrane. Migration of cells from serum free media towards serum free media (SF - SF) was used to normalize data as a control. Data are reported as median (horizontal line) with interquartile range. Statistical significance was assessed by performing a parametric one-tailed t-test with Welch's correction (unpaired); *= P ≤ 0.05; **= P ≤ 0.01; ***= P ≤ 0.001; ****= P ≤ 0.0001. **(b)** To verify the capability of cells to migrate, we performed a control experiment by adding 1% FBS medium in the lower compartment (violet points) and use migration toward SF medium as control. Furthermore, to exclude purely chemokinetic or proliferative effects we performed the experiments Effec by adding the conditioned medium (24, 48 and 72 hours) in the upper compartment and the serum free medium in the lower compartment. We obtained that indeed the conditioned media has a genuine effect as its presence in the upper chamber did not induce migration as in the previous conditions, or at least not to the same extent (blu-purple points versus yellow-orange points). Statistical significance between the two conditions was assessed by performing a parametric one-tailed t-test with Welch's correction (unpaired); *= P ≤ 0.05; Conditioned media are not able to attract cells. **= P

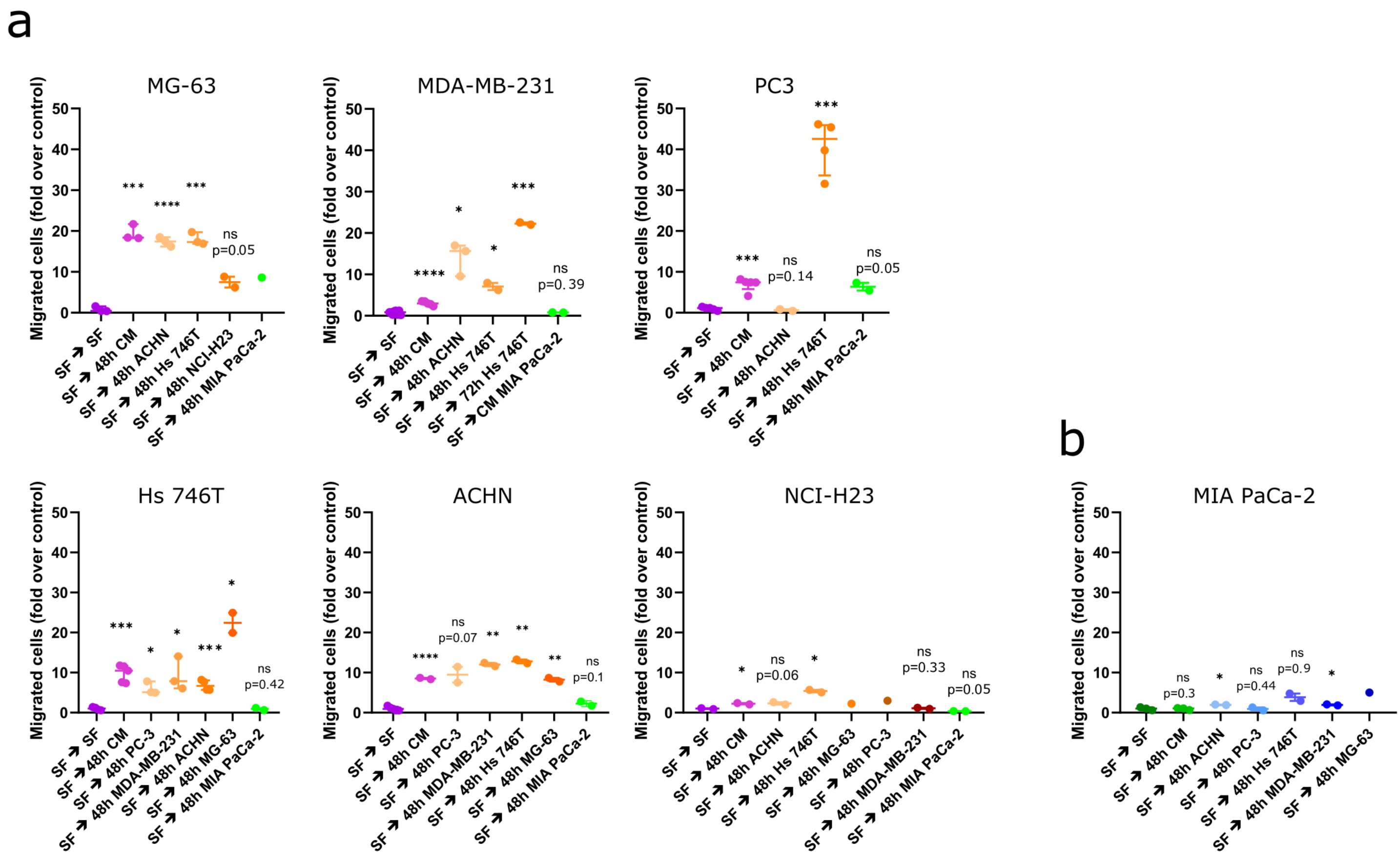

Supplementary figure 8: Cross-reactivity of conditioned media (a,b) Transwell assays performed to test whether the conditioned medium of each of the 6 aggregating cell lines, and MIA PaCa-2 as representative example of a non-aggregating cell line, act as chemoattractant for all the other cell lines. The plots report the migrated cells (fold over control) of different experiments. Each point on the plot represents a whole membrane. Data are reported as median (horizontal line) with interquartile range. Statistical significance was assessed by performing a parametric one-tailed t-test with Welch's correction (unpaired); *= P ≤ 0.05; **= P ≤ 0.01; ***= P ≤ 0.001; ****= P ≤ 0.0001.

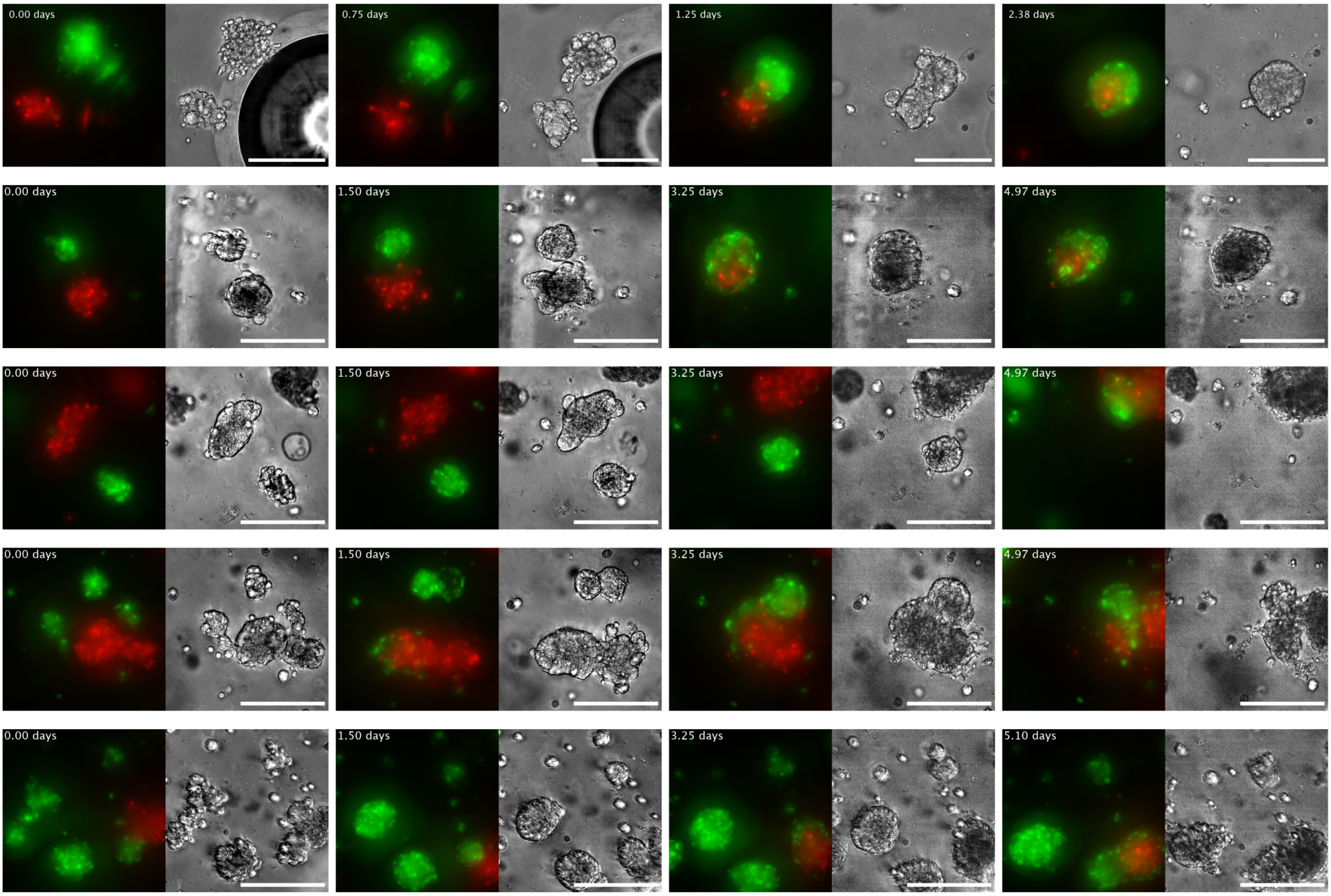

Supplementary figure 9: Heteroclonal aggregation of breast cancer cell lines Two breast cancer cell lines, MDA-MB-231 (H2B-GFP) and MCF-7 (H2B-RFP), were preassembled into spheroids and cocultured in a 1:1 ratio and were imaged over several days to record aggregation events. Heterotypic spheroids are formed over time, demonstrating the ability of these two cell lines to form heteroclonal spheroids. Each row of the figure represents a different event, where time corresponding to each snapshots are indicated in the top left corner of each image. MCF-7 were cultured in RPMI-1640.

Cell line name	ExPASy Cellosaurus RRID	Site of origin	Culture medium
**786-O**	CVCL_1051	Renal cell carcinoma	RPMI
**A-375**	CVCL_0132	Amelanotic melanoma	DMEM
**A-431**	CVCL_0037	Skin squamous cell carcinoma	DMEM
**A-498**	CVCL_1056	Renal cell carcinoma	RPMI
**A549**	CVCL_0023	Lung adenocarcinoma	RPMI
**ACHN**	CVCL_1067	Papillary renal cell carcinoma; derived from metastatic site: pleural effusion	RPMI
**BxPC-3**	CVCL_0186	Pancreatic ductal adenocarcinoma	RPMI
**Capan-1**	CVCL_0237	Pancreatic ductal adenocarcinoma; derived from metastatic site: liver	ISCOVE 20%
**CFPAC-1**	CVCL_1119	Pancreatic ductal adenocarcinoma; derived from metastatic site: liver	RPMI
**COV-362**	CVCL_2420	High grade ovarian serous adenocarcinoma; derived from metastatic site: pleural effusion	DMEM
**DLD-1**	CVCL_0248	Colon adenocarcinoma	RPMI
**DU145**	CVCL_0105	Prostate carcinoma; derived from metastatic site: brain	RPMI
**GP2d**	CVCL_2450	Colon adenocarcinoma	DMEM
**HCT 116**	CVCL_0291	Colon adenocarcinoma	RPMI
**HCT-15**	CVCL_0292	Colon adenocarcinoma	RPMI
**HOP-62**	CVCL_1285	Lung adenocarcinoma	RPMI
**Hs 746T**	CVCL_0333	Gastric adenocarcinoma; derived from metastatic site: muscle; left leg	DMEM
**HT-29**	CVCL_A8EZ	Colon adenocarcinoma	DMEM
**LNCaP**	CVCL_0395	Prostate carcinoma; derived from metastatic site: left supraclavicular lymph node	RPMI
**MDA-MB-231**	CVCL_0062	Breast adenocarcinoma; derived from metastatic site: pleural effusion	DMEM
**MG-63**	CVCL_0426	Juxtacortical osteogenic sarcoma	DMEM
**MIA PaCa2**	CVCL_0428	Pancreatic ductal adenocarcinoma	RPMI
**NCI-H1299**	CVCL_0060	Lung large cell carcinoma; derived from metastatic site: lymph node	RPMI
**NCI-H23**	CVCL_1547	Lung adenocarcinoma	RPMI
**OVCAR-8**	CVCL_1629	High grade ovarian serous adenocarcinoma	RPMI
**PANC-1**	CVCL_0480	Pancreatic ductal adenocarcinoma	RPMI
**PC-3**	CVCL_0035	Prostate carcinoma; derived from metastatic site: bone	RPMI
**SK-OV-3**	CVCL_0532	Ovarian serous cystadenocarcinoma; derived from metastatic site: ascites	McCoy's 5A
**SW48**	CVCL_1724	Colon adenocarcinoma	DMEM
**U-2 OS**	CVCL_L856	Osteosarcoma	McCoy's

Table1: list of cell lines used in this work, their tissues of origin and their culture media.

LIGAND	RECEPTOR	REFERENCES
CXCL5	CXCR1	[1, 2]
	CXCR2	[2-4]
CXCL6	CXCR1	[5, 6]
	CXCR2	[2, 7]
CXCL1	CXCR1, CXCR2	[8, 9]
CXCL8	CXCR1	[5, 6]
	CXCR2	[2]
CCL2	CCR2	[10]
CCL20	CCR6	[11, 12]
CCL20	CCR11	[13]
CCL5	CCR1, CCR3, CCR5, CCR4	[14-16]
VEGFA	FLT	[17-19]
	KDR	[18-20]
AGT	AT1, AT2	[21]
DKK1	LPR5, LPR6	[22]
GDF15	GFRAL	[23]
MDK	ALK	[24]
	NOTCH2	[25]
	SDC3	[26]
CCN3	NOTCH1	[27]
	ITGAV	[28]
	ITGA5	[28, 29]
PDGFA	PDGFRA	[30]
GRN	TNFRSF1A, TNFRSF1B	[31, 32]
	DLK1	[33, 34]
	EPHA2	[35]
	SORT1	[34, 36, 37]
IL11	IL6ST	[38, 39]
	IL11RA	[40, 41]
IL6	IL6R	[42, 43]
	IL6ST	[38, 44-46]
ANGPT1	TEK	[47-49]
HGF	MET	[50, 51]
CXCL10	CXCR3	[52, 53]
IL7	IL7R	[54]
XCL1	XCR1	[55-57]
PF4	CXCR3	[58-60]

1. Moussouras, N.A., et al., *Differences in Sulfotyrosine Binding amongst CXCR1 and CXCR2 Chemokine Ligands.* Int J Mol Sci, 2017. **18**(9).
2. Zlotnik, A. and O. Yoshie, *Chemokines: a new classification system and their role in immunity.* Immunity, 2000. **12**(2): p. 121-7.
3. Persson, T., et al., *Expression of the neutrophil-activating CXC chemokine ENA-78/CXCL5 by human eosinophils.* Clin Exp Allergy, 2003. **33**(4): p. 531-7.
4. Romero-Moreno, R., et al., *The CXCL5/CXCR2 axis is sufficient to promote breast cancer colonization during bone metastasis.* Nat Commun, 2019. **10**(1): p. 4404.
5. Wolf, M., et al., *Granulocyte chemotactic protein 2 acts via both IL-8 receptors, CXCR1 and CXCR2.* Eur J Immunol, 1998. **28**(1): p. 164-70.

6. Ahuja, S.K. and P.M. Murphy, *The CXC chemokines growth-regulated oncogene (GRO) alpha, GRObeta, GROgamma, neutrophil-activating peptide-2, and epithelial cell-derived neutrophil-activating peptide-78 are potent agonists for the type B, but not the type A, human interleukin-8 receptor.* J Biol Chem, 1996. **271**(34): p. 20545-50.
7. Liu, G., et al., *Activation of CXCL6/CXCR1/2 Axis Promotes the Growth and Metastasis of Osteosarcoma Cells.* Front Pharmacol, 2019. **10**: p. 307.
8. Korbecki, J., et al., *: Gene, Promoter, Regulation of Expression, mRNA Stability, Regulation of Activity in the Intercellular Space.* Int J Mol Sci, 2022. **23**(2).
9. Korbecki, J., et al., *CXCR2 Receptor: Regulation of Expression, Signal Transduction, and Involvement in Cancer.* Int J Mol Sci, 2022. **23**(4).
10. Hao, Q., J.V. Vadgama, and P. Wang, *CCL2/CCR2 signaling in cancer pathogenesis.* Cell Commun Signal, 2020. **18**(1): p. 82.
11. Schutyser, E., S. Struyf, and J. Van Damme, *The CC chemokine CCL20 and its receptor CCR6.* Cytokine Growth Factor Rev, 2003. **14**(5): p. 409-26.
12. Kadomoto, S., K. Izumi, and A. Mizokami, *The CCL20-CCR6 Axis in Cancer Progression.* Int J Mol Sci, 2020. **21**(15).
13. Matti, C., et al., *CCL20 is a novel ligand for the scavenging atypical chemokine receptor 4.* J Leukoc Biol, 2020. **107**(6): p. 1137-1154.
14. Appay, V. and S.L. Rowland-Jones, *RANTES: a versatile and controversial chemokine.* Trends Immunol, 2001. **22**(2): p. 83-7.
15. Schall, T.J., *Biology of the RANTES/SIS cytokine family.* Cytokine, 1991. **3**(3): p. 165-83.
16. Hadida, F., et al., *HIV-specific T cell cytotoxicity mediated by RANTES via the chemokine receptor CCR3.* J Exp Med, 1998. **188**(3): p. 609-14.
17. Roberts, D.M., et al., *The vascular endothelial growth factor (VEGF) receptor Flt-1 (VEGFR-1) modulates Flk-1 (VEGFR-2) signaling during blood vessel formation.* Am J Pathol, 2004. **164**(5): p. 1531-5.
18. Neufeld, G., et al., *Vascular endothelial growth factor (VEGF) and its receptors.* FASEB J, 1999. **13**(1): p. 9-22.
19. Matsumoto, T. and L. Claesson-Welsh, *VEGF receptor signal transduction.* Sci STKE, 2001. **2001**(112): p. re21.
20. Li, B., et al., *KDR (VEGF receptor 2) is the major mediator for the hypotensive effect of VEGF.* Hypertension, 2002. **39**(6): p. 1095-100.
21. Arrieta, O., et al., *Expression of AT1 and AT2 angiotensin receptors in astrocytomas is associated with poor prognosis.* Br J Cancer, 2008. **99**(1): p. 160-6.
22. Cheng, Z., et al., *Crystal structures of the extracellular domain of LRP6 and its complex with DKK1.* Nat Struct Mol Biol, 2011. **18**(11): p. 1204-10.
23. Yang, L., et al., *GFRAL is the receptor for GDF15 and is required for the anti-obesity effects of the ligand.* Nat Med, 2017. **23**(10): p. 1158-1166.
24. Stoica, G.E., et al., *Midkine binds to anaplastic lymphoma kinase (ALK) and acts as a growth factor for different cell types.* J Biol Chem, 2002. **277**(39): p. 35990-8.
25. Huang, Y., et al., *Midkine induces epithelial-mesenchymal transition through Notch2/Jak2-Stat3 signaling in human keratinocytes.* Cell Cycle, 2008. **7**(11): p. 1613-22.
26. Kurosawa, N., et al., *Glypican-2 binds to midkine: the role of glypican-2 in neuronal cell adhesion and neurite outgrowth.* Glycoconj J, 2001. **18**(6): p. 499-507.
27. Sakamoto, K., et al., *The nephroblastoma overexpressed gene (NOV/ccn3) protein associates with Notch1 extracellular domain and inhibits myoblast differentiation via Notch signaling pathway.* J Biol Chem, 2002. **277**(33): p. 29399-405.
28. Lin, C.G., et al., *CCN3 (NOV) is a novel angiogenic regulator of the CCN protein family.* J Biol Chem, 2003. **278**(26): p. 24200-8.
29. Lin, C.G., et al., *Integrin-dependent functions of the angiogenic inducer NOV (CCN3): implication in wound healing.* J Biol Chem, 2005. **280**(9): p. 8229-37.
30. Chen, P.H., X. Chen, and X. He, *Platelet-derived growth factors and their receptors: structural and functional perspectives.* Biochim Biophys Acta, 2013. **1834**(10): p. 2176-86.

31. Tang, W., et al., *The growth factor progranulin binds to TNF receptors and is therapeutic against inflammatory arthritis in mice.* Science, 2011. **332**(6028): p. 478-84.
32. Williams, A., et al., *Review: Novel Insights Into Tumor Necrosis Factor Receptor, Death Receptor 3, and Progranulin Pathways in Arthritis and Bone Remodeling.* Arthritis Rheumatol, 2016. **68**(12): p. 2845-2856.
33. Baladrón, V., et al., *The EGF-like homeotic protein dlk affects cell growth and interacts with growth-modulating molecules in the yeast two-hybrid system.* Biochem Biophys Res Commun, 2002. **291**(2): p. 193-204.
34. Cui, Y., A. Hettinghouse, and C.J. Liu, *Progranulin: A conductor of receptors orchestra, a chaperone of lysosomal enzymes and a therapeutic target for multiple diseases.* Cytokine Growth Factor Rev, 2019. **45**: p. 53-64.
35. Neill, T., et al., *EphA2 is a functional receptor for the growth factor progranulin.* J Cell Biol, 2016. **215**(5): p. 687-703.
36. Kawashima, K.I., et al., *Glucose deprivation regulates the progranulin-sortilin axis in PC12 cells.* FEBS Open Bio, 2017. **7**(2): p. 149-159.
37. Hu, F., et al., *Sortilin-mediated endocytosis determines levels of the frontotemporal dementia protein, progranulin.* Neuron, 2010. **68**(4): p. 654-67.
38. Murakami, M., D. Kamimura, and T. Hirano, *Pleiotropy and Specificity: Insights from the Interleukin 6 Family of Cytokines.* Immunity, 2019. **50**(4): p. 812-831.
39. Kurth, I., et al., *Activation of the signal transducer glycoprotein 130 by both IL-6 and IL-11 requires two distinct binding epitopes.* J Immunol, 1999. **162**(3): p. 1480-7.
40. Balakrishnan, L., et al., *IL-11/IL11RA receptor mediated signaling: a web accessible knowledgebase.* Cell Commun Adhes, 2013. **20**(3-4): p. 81-6.
41. Yang, Y.C. and T. Yin, *Interleukin-11 and its receptor.* Biofactors, 1992. **4**(1): p. 15-21.
42. Rose-John, S., *IL-6 trans-signaling via the soluble IL-6 receptor: importance for the pro-inflammatory activities of IL-6.* Int J Biol Sci, 2012. **8**(9): p. 1237-47.
43. Wolf, J., S. Rose-John, and C. Garbers, *Interleukin-6 and its receptors: a highly regulated and dynamic system.* Cytokine, 2014. **70**(1): p. 11-20.
44. Benrick, A., et al., *A non-conservative polymorphism in the IL-6 signal transducer (IL6ST)/gp130 is associated with myocardial infarction in a hypertensive population.* Regul Pept, 2008. **146**(1-3): p. 189-96.
45. Boulanger, M.J. and K.C. Garcia, *Shared cytokine signaling receptors: structural insights from the gp130 system.* Adv Protein Chem, 2004. **68**: p. 107-46.
46. Kallen, K.J., *The role of transsignalling via the agonistic soluble IL-6 receptor in human diseases.* Biochim Biophys Acta, 2002. **1592**(3): p. 323-43.
47. Souma, T., et al., *Angiopoietin receptor TEK mutations underlie primary congenital glaucoma with variable expressivity.* J Clin Invest, 2016. **126**(7): p. 2575-87.
48. Thurston, G. and C. Daly, *The complex role of angiopoietin-2 in the angiopoietin-tie signaling pathway.* Cold Spring Harb Perspect Med, 2012. **2**(9): p. a006550.
49. Jeansson, M., et al., *Angiopoietin-1 is essential in mouse vasculature during development and in response to injury.* J Clin Invest, 2011. **121**(6): p. 2278-89.
50. Naldini, L., et al., *Scatter factor and hepatocyte growth factor are indistinguishable ligands for the MET receptor.* EMBO J, 1991. **10**(10): p. 2867-78.
51. Bottaro, D.P., et al., *Identification of the hepatocyte growth factor receptor as the c-met proto-oncogene product.* Science, 1991. **251**(4995): p. 802-4.
52. Pandey, V., et al., *CXCL10/CXCR3 signaling contributes to an inflammatory microenvironment and its blockade enhances progression of murine pancreatic precancerous lesions.* Elife, 2021. **10**.
53. Brightling, C.E., et al., *The CXCL10/CXCR3 axis mediates human lung mast cell migration to asthmatic airway smooth muscle.* Am J Respir Crit Care Med, 2005. **171**(10): p. 1103-8.
54. Al-Rawi, M.A., R.E. Mansel, and W.G. Jiang, *Interleukin-7 (IL-7) and IL-7 receptor (IL-7R) signalling complex in human solid tumours.* Histol Histopathol, 2003. **18**(3): p. 911-23.
55. Lei, Y. and Y. Takahama, *XCL1 and XCR1 in the immune system.* Microbes Infect, 2012. **14**(3): p. 262-7.

56. Yamazaki, C., et al., *Conservation of a chemokine system, XCR1 and its ligand, XCL1, between human and mice.* Biochem Biophys Res Commun, 2010. **397**(4): p. 756-61.
57. Kroczek, R.A. and V. Henn, *The Role of XCR1 and its Ligand XCL1 in Antigen Cross-Presentation by Murine and Human Dendritic Cells.* Front Immunol, 2012. **3**: p. 14.
58. Mueller, A., et al., *CXCL4-induced migration of activated T lymphocytes is mediated by the chemokine receptor CXCR3.* J Leukoc Biol, 2008. **83**(4): p. 875-82.
59. Deng, S., et al., *Non-platelet-derived CXCL4 differentially regulates cytotoxic and regulatory T cells through CXCR3 to suppress the immune response to colon cancer.* Cancer Lett, 2019. **443**: p. 1-12.
60. Wang, X., et al., *Effects of CXCL4/CXCR3 on the lipopolysaccharide-induced injury in human umbilical vein endothelial cells.* J Cell Physiol, 2019. **234**(12): p. 22378-22385.

Table2: references providing evidence of receptor activity to found ligands.

Supplementary movie legends

SI Movie 1: Single cell seeded MDA-MB-231. MDA-MB-231 single cells embedded in a Matrigel and imaged once a day for 16 days after seeding. Seeding density *a posteriori*: 22,2 cells/mm^3. Scale bar: 200 µm.

SI Movie 2: Single cell seeded PANC1. PANC1 single cells embedded in a Matrigel and imaged once a day for 15 days after seeding. This cell line is reported as representative of the behaviour of non-aggregating cell lines. Seeding density *a posteriori:* 59,5 cells/mm^3. Scale bar: 200 µm.

SI Movie 3: Pre-formed spheroids of MDA-MB-231. Pre-formed spheroids of MDA-MB-231 embedded in Matrigel and imaged every 2 hours for 4 days. Seeding density: 2,5 spheroids/mm^3. Scale bar: 200 µm.

SI Movie 4: Pre-formed spheroids of MDA-MB-231 treated with mitomycin. Pre-formed spheroids of MDA-MB-231 embedded in Matrigel and treated with 0.75 µg/ml mitomycin. Seeding density: 2,5 spheroids/mm^3. Scale bar: 200 µm.

SI Movie 5: Pre-formed spheroids of PC-3 treated with mitomycin. Pre-formed spheroids of PC-3 embedded in Matrigel and treated with 0.75 µg/ml mitomycin. Seeding density: 2,5 spheroids/mm^3. Scale bar: 200 µm.

SI Movie 6: Pre-formed spheroids of MG-63 treated with mitomycin. Pre-formed spheroids of MG-63 embedded in Matrigel and treated with 0.75 µg/ml mitomycin. Seeding density: 2,5 spheroids/mm^3. Scale bar: 200 µm.

SI Movie 7: Pre-formed spheroids of PC-3 expressing fluorescent LifeAct. Pre-formed spheroids of MG-63 cells transduced with LifeAct-GFP (green) or LifeAct-Ruby (red) expressing lentiviral vectors, embedded in Matrigel and imaged every hour for 3 days. Scale bar: 100 µm.

SI Movie 8: Pre-formed spheroids of MDA-MB-231 expressing fluorescent LifeAct. Pre-formed spheroids of LifeAct-Ruby expressing MDA-MB-231 embedded in Matrigel and imaged every 30 minutes for two days. Scale bar: 100 µm.

SI Movie 9: High-temporal resolution timelapse of pre-formed spheroids of MDA-MB-231. Pre-formed spheroids of MDA-MB-231 embedded in Matrigel and imaged every 45 minutes for 4 days. Seeding density: 2,5 spheroids/mm^3. Scale bar: 200 µm.

SI Movie 10: Pre-formed spheroids of MDA-MB-231 treated with Latrunculin A. Pre-formed spheroids of MDA-MB-231 embedded in Matrigel, treated with 1 µm Latrunculin A and imaged every 45 minutes for 4 days. Seeding density: 2,5 spheroids/mm^3. Scale bar: 200 µm.

SI Movie 11: Pre-formed spheroids of MDA-MB-231 treated with Blebbistatin. Pre-formed spheroids of MDA-MB-231 embedded in Matrigel, treated with 100 µm Blebbistatin and imaged every 45 minutes for 4 days. Seeding density: 2,5 spheroids/mm^3. Scale bar: 200 µm.

SI Movie 12: Pre-formed spheroids of MDA-MB-231 treated with Y-27632. Pre-formed spheroids of MDA-MB-231 embedded in Matrigel, treated with 20 µM Y-27632 and imaged every 45 minutes for 4 days. Seeding density: 2,5 spheroids/mm^3. Scale bar: 200 µm.

SI Movie 13: Pre-formed spheroids of MDA-MB-231 treated with Wiskostatin. Pre-formed spheroids of MDA-MB-231 embedded in Matrigel, treated with 10 µM Wiskostatin and imaged every 45 minutes for 4 days. Seeding density: 2,5 spheroids/mm^3. Scale bar: 200 µm.

SI Movie 14: Pre-formed spheroids of MDA-MB-231 treated with CK666. Pre-formed spheroids of MDA-MB-231 embedded in Matrigel, treated with 10o µM CK666 and imaged every 45 minutes for 4 days. Seeding density: 2,5 spheroids/mm^3. Scale bar: 200 µm.

SI Movie 15: Pre-formed spheroids of MDA-MB-231 treated with Cytochalasin D. Pre-formed spheroids of MDA-MB-231 embedded in Matrigel, treated with 1 µM Cytochalasin D and imaged every 45 minutes for 4 days. Seeding density: 2,5 spheroids/mm^3. Scale bar: 200 µm.

SI Movie 16: Pre-formed spheroids of MDA-MB-231 treated with BEZ235. Pre-formed spheroids of MDA-MB-231 embedded in Matrigel, treated with BEZ235 100 nM and imaged every 45 minutes for 4 days. Seeding density: 2,5 spheroids/mm^3. Scale bar: 200 µm.

SI Movie 17: Pre-formed spheroids of MDA-MB-231 treated with BYL719. Pre-formed spheroids of MDA-MB-231 embedded in Matrigel, treated with BYL719 3 µM and imaged every 45 minutes for 4 days. Seeding density: 2,5 spheroids/mm^3. Scale bar: 200 µm.

SI Movie 18: Pre-formed spheroids of MDA-MB-231 treated with AZD-6244. Pre-formed spheroids of MDA-MB-231 embedded in Matrigel, treated with AZD-6244 0,5 µM and imaged every 2 hours for 4 days. Seeding density: 2,5 spheroids/mm^3. Scale bar: 200 µm.

SI Movie 19: Pre-formed spheroids of MDA-MB-231 treated with MK-2206. Pre-formed spheroids of MDA-MB-231 embedded in Matrigel, treated with MK-2206 5 µM and imaged every 45 minutes for 4 days. Seeding density: 2,5 spheroids/mm^3. Scale bar: 200 µm.

SI Movie 20: Heteroclonal aggregation of MDA-MB-231 and PC-3 fluorescently tagged with H2B. Pre-formed spheroids of H2B-RFP MDA-MB-231 (red) and H2B-GFP PC-3 (green) seeded in Matrigel. Spheroids were imaged every 2 hours for 4 days. Scale bar: 200 µm.

SI Movie 21: Heteroclonal aggregation of MDA-MB-231 and MG-63 fluorescently tagged with H2B. Pre-formed spheroids of H2B-RFP MDA-MB-231 (red) and H2B-GFP MG-63 (green) cell lines seeded in Matrigel. Spheroids were imaged every 2 hours for 4 days. Scale bar: 200 µm.

SI Movie 22: Heteroclonal aggregation of MG-63 and PC-3 fluorescently tagged with H2B. Pre-formed spheroids of H2B-RFP PC-3 (red) and H2B-GFP MG-63 (green) cell lines seeded in Matrigel. Spheroids were imaged every 2 hours for 4 days. Scale bar: 200 µm.

SI Movie 23: Heteroclonal aggregation of MDA-MB-231 and PC-3 fluorescently tagged with LifeAct. Pre-formed spheroids of LifeAct-Ruby MDA-MB-231 (red) and LifeAct-GFP PC-3 (green) cell lines in Matrigel. Spheroids were imaged every hour for 2 days. Scale bar: 100 µm. Scale bar: 100 µm.
